# Supplementary material for: MPK3- and MPK6-mediated VLN3 phosphorylation regulates actin dynamics during stomatal immunity in Arabidopsis
Source: Nat Commun. 2021 Nov 9;12:6474. doi: 10.1038/s41467-021-26827-2 (PMC8578381; doi:10.1038/s41467-021-26827-2)
Supplement: Supplementary file 1 — Supplementary Information [file 41467_2021_26827_MOESM1_ESM.pdf]

# Supplementary information

| Clone | Forward Primer | Reverse Primer |
|---|---|---|
| *VLN3* | 5´CATGGACGAGCTGTACAAGGGTACCATGTCTGGGTCAACAAAAGTATTGGATC -3´ | 5´GTGGACTCCTCTTAGAATTCCCGGGTTAGAATAAGTTGAATTTCTTCTTCAGTAAGTCTTG -3´ |
| *C-VLN3* | 5´ACGGGGGACGAGCTCGGTACCATGTCTACTAAAGCTACTGTGCAAG -3´ | 5´-AACATCGTATGGGTAGTCGACGAATAAGTTGAATTTCTTCTTCA -3´ |
| *C-VLN3*$^{S779A}$ | 5´-TTCTTCTGGGAGAACAAGCGCTCCGAGCCGAGACAGATCAAATGGAAGT -3´ | 5´-ACTTCCATTTGATCTGTCTCGGCTCGGAGCGCTTGTTCTCCCAGAAGAA -3´ |
| *C-VLN3*$^{S779D}$ | 5´-TTCTTCTGGGAGAACAAGCGATCCGAGCCGAGACAGATCAAATGGAAGT-3´ | 5´-ACTTCCATTTGATCTGTCTCGGCTCGGATCGCTTGTTCTCCCAGAAGAA-3´ |
| *C-VLN3*$^{S850A}$ | 5´-CACCTGATACCGCTCCTTCTGCTGAAGCAAAAGAT-3´ | 5´-CAGCAGAAGGAGCGGTATCAGGTGATTTCTTCTTCT-3´ |
| *C-VLN3*$^{850D}$ | 5´-CACCTGATACCGATCCTTCTGCTGAAGCAAAAGAT-3´ | 5´-CAGCAGAAGGATCGGTATCAGGTGATTTCTTCTTCT-3´ |
| *C-VLN3*$^{S809A}$ | 5´-GCATTCAATTCTGCTCCATCATCAAAGTCACCTC -3´ | 5´-GATGGAGCAGAATTGAATGCGGATGTTAAGGC -3´ |
| *GST-VLN3* | 5´-GTTCTGTTCCAGGGGCCCCTGGGATCCATGTCTGGGTCAACAAAAGTATTGGATC -3´ | 5´-CGATGCGGCCGCTCGAGTCGACCCGGGTTAGAATAAGTTGAATTTCTTCTTCAGTAAGTCTTG -3´ |
| *GST-N-VLN2* | 5´-GTTCTGTTCCAGGGGCCCCTGGGATCCATGTCAACAAAAGTGTTGGATCCT-3´ | 5´-CGATGCGGCCGCTCGAGTCGACCCGGGCTAATCCCAAGAAAAGTAAGTGGTGAA-3´ |
| *GST-C-VLN2* | 5´-GTTCTGTTCCAGGGGCCCCTGGGATCCATGGCCACTAAAGCTATCGTACAAG -3´ | 5´-CGATGCGGCCGCTCGAGTCGACCCGGGCTAGAACAAGTCGAACTTCTTCTTAAGCA -3´ |
| *GST-C-VLN3* | 5´-ACGCGTCGACTAATGTCTACTAAAGCTACTGTGCAAG-3´ | 5´-ATAAGAATGCGGCCGCTTAGAATAAGTTGAATTTCTTCTTCA-3´ |
| *GST-N-VLN3* | 5´-ACGCGTCGACTAATGTCTGGGTCAACAAAAGTATT-3´ | 5´-ATAAGAATGCGGCCGCTTAATCCCATGAAAAATAAGTGG -3´ |

**Supplementary Table 1. DNA primers used for the cloning of various constructs described in this study.**

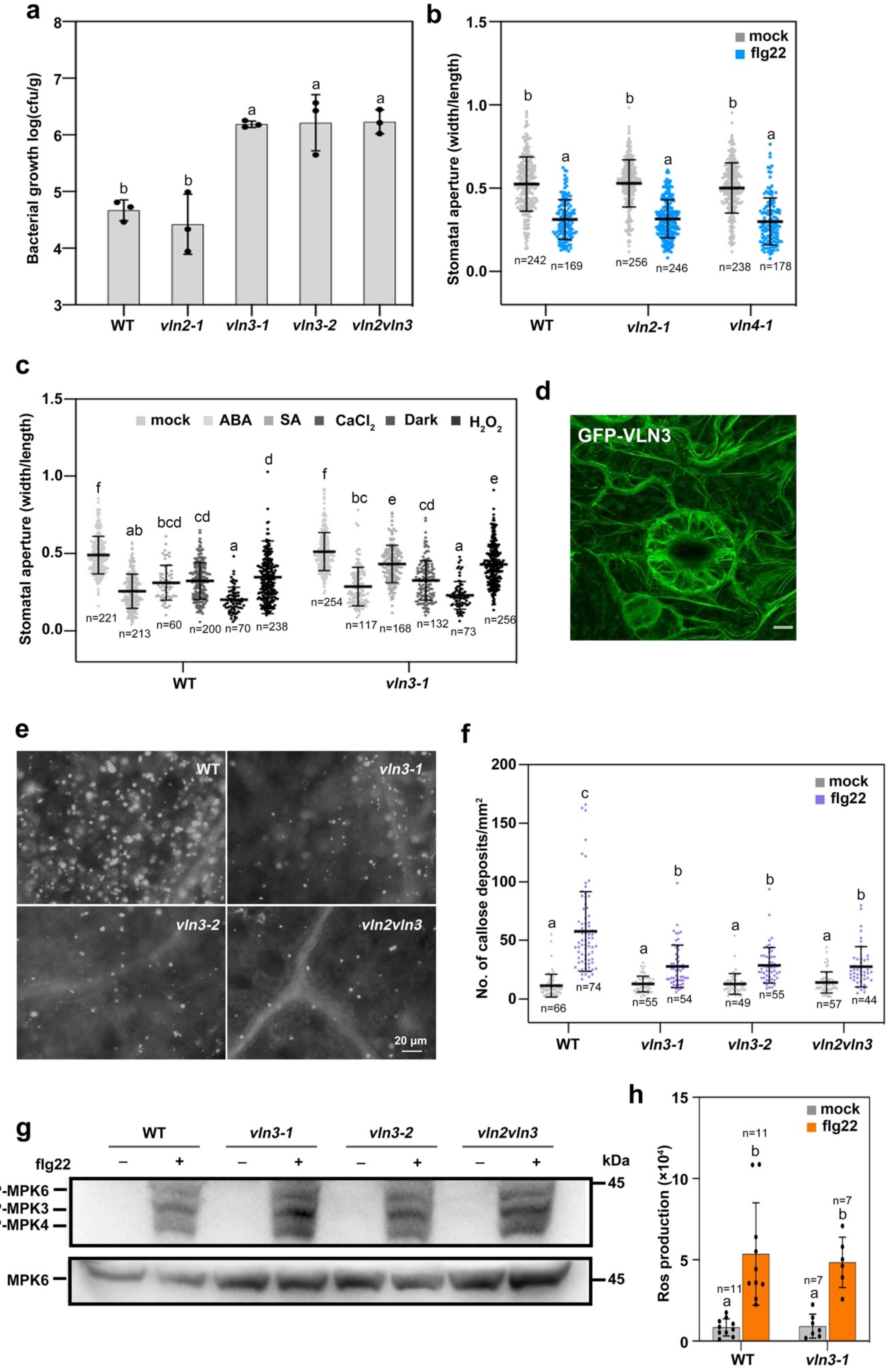


**Supplementary Figure 1. PTI responses in the *vln3* mutant.**

(a) Bacterial growth was examined in WT, *vln2-1*, *vln3-1*, *vln3-2* and *vln2vln3* mutants. Plants of indicated genotypes were sprayed with DC3000. Bacterial growth was measured at 2 dpi. (b) Stomatal aperture in different *vln* mutants treated with flg22. (c) Stomatal aperture was measured in epidermal peels treated with mock, 100 µM SA, 10 µM ABA, 100 µM $CaCl_2$, 100 µM $H_2O_2$ or dark for 1 hr. (d) Localization of GFP-VLN3 expressed from the native VLN3 promoter. Scale bar, 10 µm. (e,f) Number of callose deposits indicated by aniline blue-stained spots was quantified in WT and mutants following mock, 1 µM flg22 treatment for 24 hr. (g) MAPK activation in WT and mutants in response to treatment with 1 µM flg22 for 10 min. Total protein extracts were probed with anti-MPK6 or anti-p44/42 MAPK to assess MPK6 protein levels or phosphorylation of MPK3, MPK4 and MPK6, respectively. (h) Apoplastic ROS production was measured on 4-week-old leaf disks upon 1 µM flg22 treatment using a luminescence assay. Photon counts at peaks were shown in WT and mutant. Value are means ± SD. n=3 for bacterial growth measurements. Sample sizes in (b,c,f,h) are indicated. Different letters indicate significant differences at $P<0.05$, as determined by two-way ANOVA with Tukey's multiple comparisons test. The exact p-values are provided in the Source Data file.

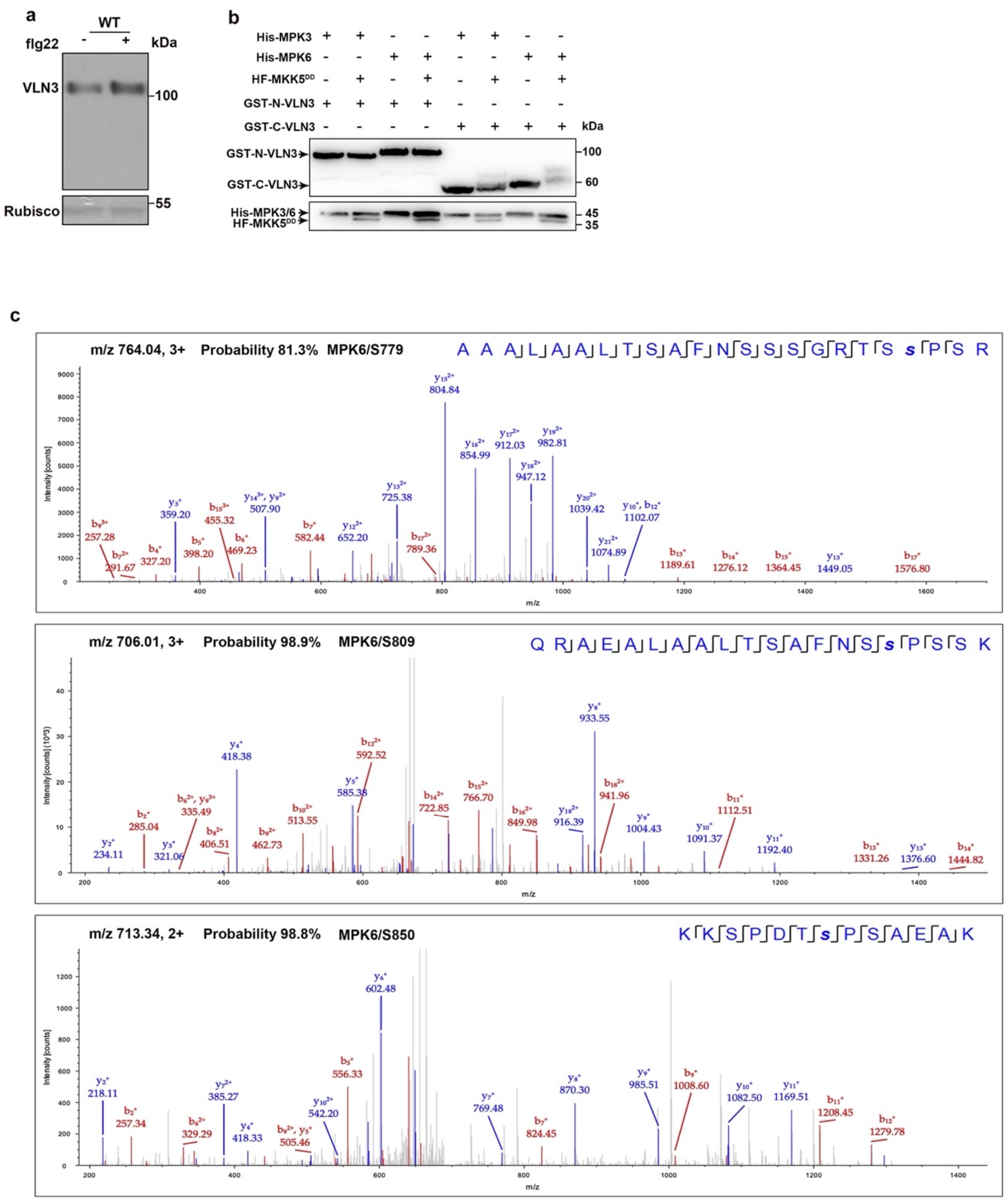


**Supplementary Figure 2. Phosphosites identification.**

(a) Full-length VLN3 tagged with HA was expressed in Arabidopsis protoplasts. Following treatment with mock or 100 nM flg22 for 10 min, total protein was separated in a phos-tag gel and VLN3 was detected by anti-HA antibodies. Rubisco was used as a loading control. (b) Activated MPK3 and MPK6 phosphorylate C terminus of VLN3 in vitro. The N- and C-terminal fragments of VLN3 were incubated with MPK3 or MPK6 in an in vitro kinase assay. The phosphorylation of VLN3 was detected in a phos-tag gel with anti-GST antibodies. MKK5$^{DD}$ and MPK3/6 was detected in SDS-PAGE using

anti-His antibodies. (c) Mass spectra and identification of VLN3 Ser779, Ser809, Ser850 phosphorylation by MPK6. The experiments in (a) were repeated twice, and (b) three times with similar results.

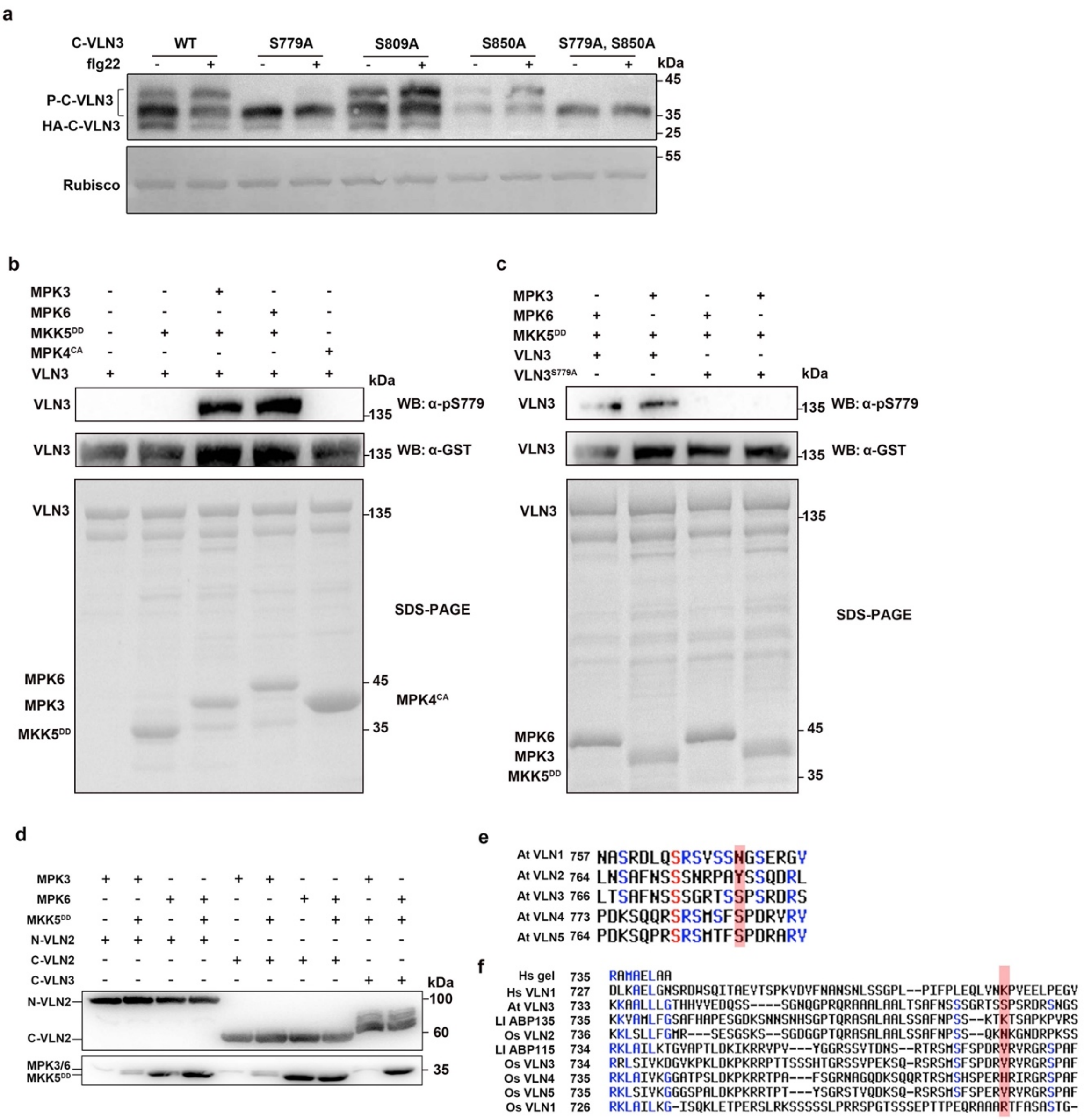


**Supplementary Figure 3. Ser779 is required for MPK3/6-mediated VLN3 phosphorylation upon flg22 treatment.**

(a) HA-tagged WT and mutated C-VLN3 were expressed in Arabidopsis protoplasts. Following treatment with mock or 100 nM flg22 for 10 min, total protein was separated in a phos-tag gel and VLN3 fragments were detected using anti-HA antibodies. Rubisco was used as a loading control. (b) Phospho-antibodies for VLN3 pS779 detect VLN3 phosphorylation by MPK3/6, no signal was detected when VLN3 was incubated with MPK4$^{CA}$ or MKK5$^{DD}$. (c) Anti-pS779 specifically detect WT VLN3, but not phosphorylation null VLN3$^{S779A}$. (d) VLN2 is not phosphorylated by MPK3/6. An in vitro kinase assay was performed by incubating the N- and C-terminal fragments of VLN2 (N-VLN2: 1-718 amino acids; C-VLN2: 719-976 amino acids), or C-VLN3 with

activated MPK3 or MPK6. Proteins were separated in a phos-tag gel. VLN proteins were detected with anti-GST antibodies. $MKK5^{DD}$ and MPK3/6 was detected in SDS-PAGE using anti-His antibodies. VLN2 fragments did not show mobility shift as C-VLN3 did when incubated with activated MPK3/6. (e) Sequence alignment of Arabidopsis villins showed that Ser779 in VLN3 also exists in VLN4 and VLN5, but not in VLN1 and VLN2 (red highlighting). (f) Sequence alignment of Arabidopsis VLN3 and villin-like proteins from rice, lily and human. Ser779 (red highlighting) is not conserved in plant and human villins. The alignment was performed by MultiAlin algorithm. Amino acid coordinates are indicated on the left. The experiments in (a-c) were repeated twice, and (d) three times with similar results.

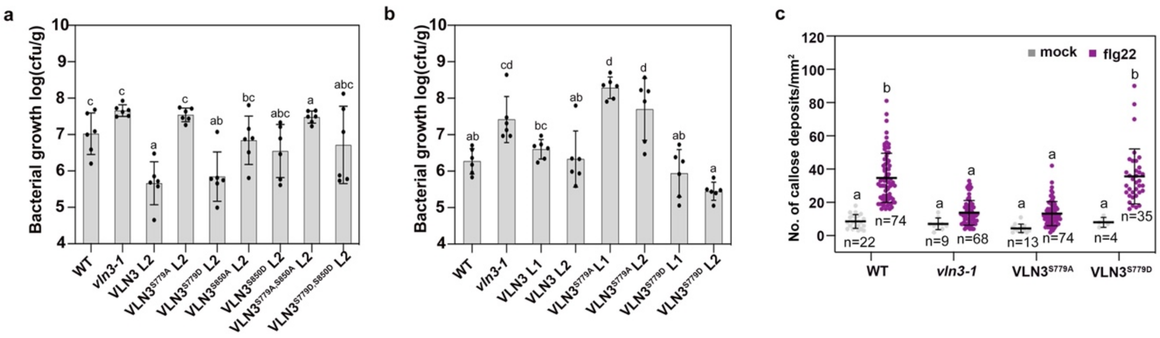


**Supplementary Figure 4. VLN3 phosphorylation is involved in both stomatal and apoplastic defenses.**

(a) Bacterial growth was determined on the second *vln3* complementary lines by spray inoculation. (b) Bacterial growth was determined on indicated plants by infiltration inoculation. L1 and L2 indicate two independent transgenic lines. (c) Quantification of flg22-induced callose deposits on WT, *vln3* mutant and *vln3* mutant expressing $\text{VLN3}^{\text{S779A}}$, $\text{VLN3}^{\text{S779D}}$. Value are means ± SD. n=6 in (a), n=3 in (b) for bacterial growth measurements. The number of images measured in (c) are indicated. Different letters indicate significant differences at P<0.05, as determined by two-way ANOVA with Tukey’s multiple comparisons test. The exact p-values are provided in the Source Data file.

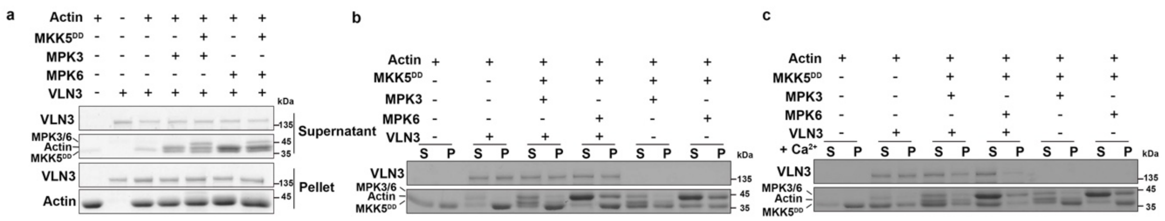


**Supplementary Figure 5. The effects of VLN3 phosphorylation on actin dynamics in vitro.**

High-speed (a, c) and low-speed (b) cosedimentation assays were used to determine the actin binding (a), bundling (b) and severing activity (c) of phosphorylated or nonphosphorylated VLN3 in vitro. Prepolymerized F-actin (3 µM) was incubated with 1 µM phosphorylated or nonphosphorylated VLN3. Actin binding and bundling assays were performed in the presence of 2 mM EGTA, and 100 µM free $Ca^{2+}$ was added in severing assays. The supernatants and pellets obtained were subjected to SDS-PAGE and Coomassie stained. The experiments in (a-c) were repeated three times with similar results.

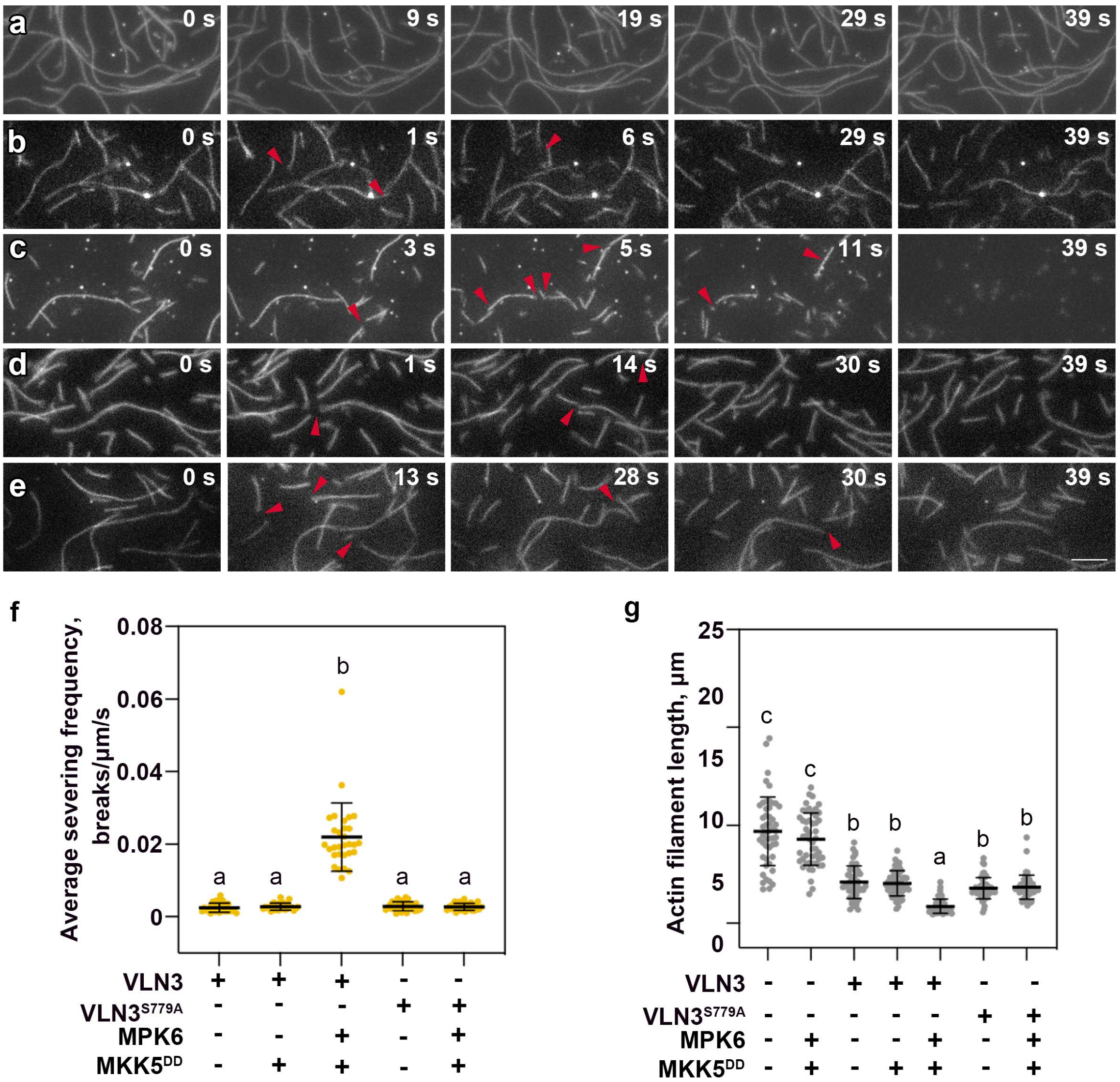


**Supplementary Figure 6. The TIRF assay shows that VLN3 phosphorylated by MPK6 exhibits enhanced $Ca^{2+}$-dependent severing activity.**

(a-e) VLN3-mediated actin filament severing was visualized by time-lapse TIRF microscopy. Oregon-green labeled actin filaments adhered to the cover slip of a perfusion chamber, and then 1 nM wild-type or mutant VLN3 in the presence of 10 µM free $Ca^{2+}$, was perfused into the chamber and time-lapse images were collected every second. Individual filaments showed breaks (arrows) along their length. The elapsed time in seconds is given in the top right corner of each image (a, actin + MPK6 + MKK5$^{DD}$; b, actin + VLN3; c, actin + VLN3 + MPK6 + MKK5$^{DD}$; d, actin + VLN3$^{S779A}$ + MPK6 + MKK5$^{DD}$; e, actin + VLN3 + MKK5$^{DD}$). Bars = 5 µm. (f) Severing frequency was calculated as the number of breaks per unit filament length per unit time. (g) Average actin filament length was measured for each indicated reaction. Value are

means ± SD. n in (f)=30, in (g)= 50 filaments from at least 5 images for each treatment. Different letters indicate significant differences at $P<0.05$, as determined by two-way ANOVA with Tukey's multiple comparisons test. The exact p-values are provided in the Source Data file.

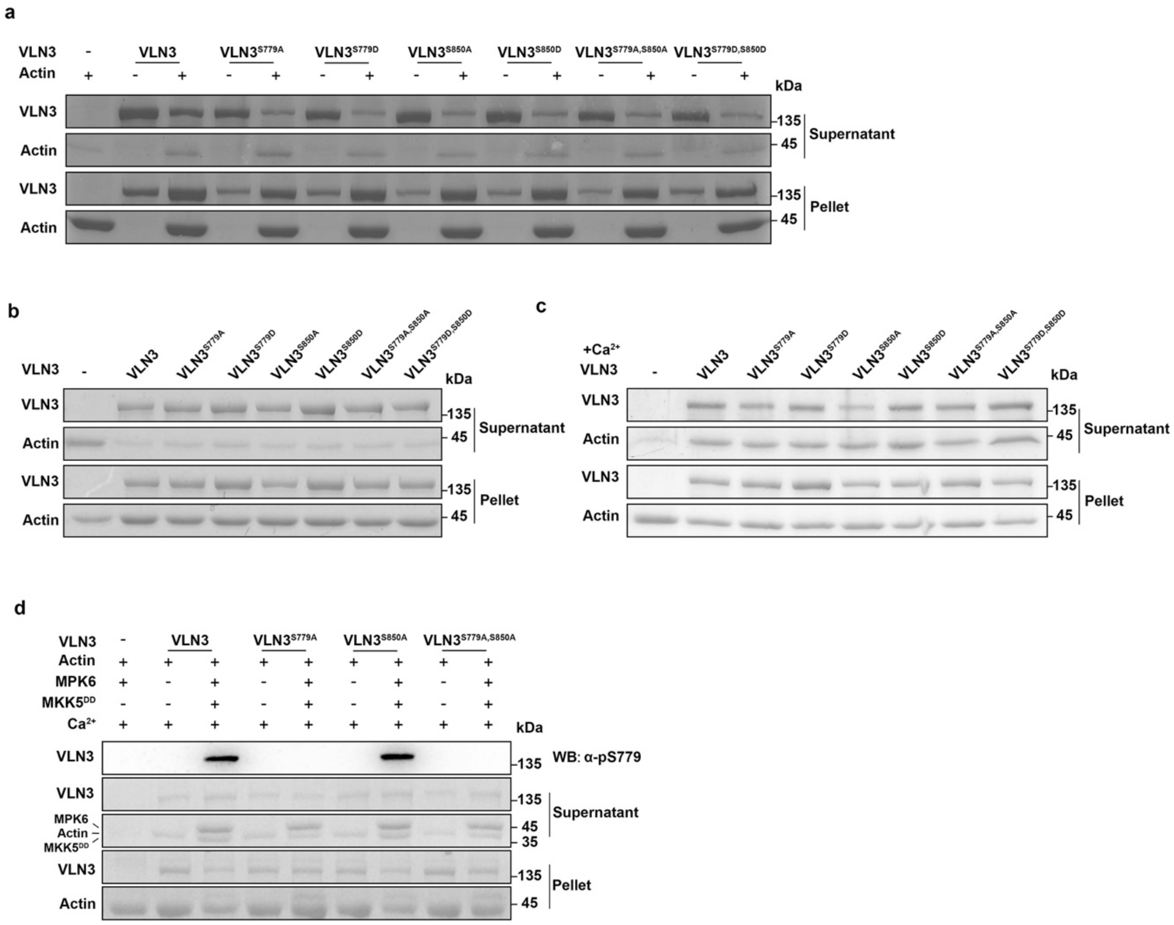


**Supplementary Figure 7. Ser779 phosphorylation is required for the enhanced filament severing activity of VLN3.**

High-speed (a, c) and low-speed (b) cosedimentation assays were used to determine the actin binding (a), bundling (b) and severing activity (c) of wild-type or mutated VLN3 in vitro. (d) High-speed cosedimentation assays were used to determine the actin severing of wild-type or mutated VLN3 in the absence or presence of activated MPK6 (d). Prepolymerized F-actin (3 µM) was incubated with 1 µM VLN3 proteins. Actin binding and bundling assays were performed in the presence of 2 mM EGTA, and 100 µM free $Ca^{2+}$ was added in severing assays. The supernatants and pellets obtained were subjected to SDS-PAGE and Coomassie stained. The reaction mixes in (d) were probed with α-pSer779 to confirm that VLN3 was phosphorylated by MPK6 in this assay. The experiments in (a-d) were repeated three times with similar results.

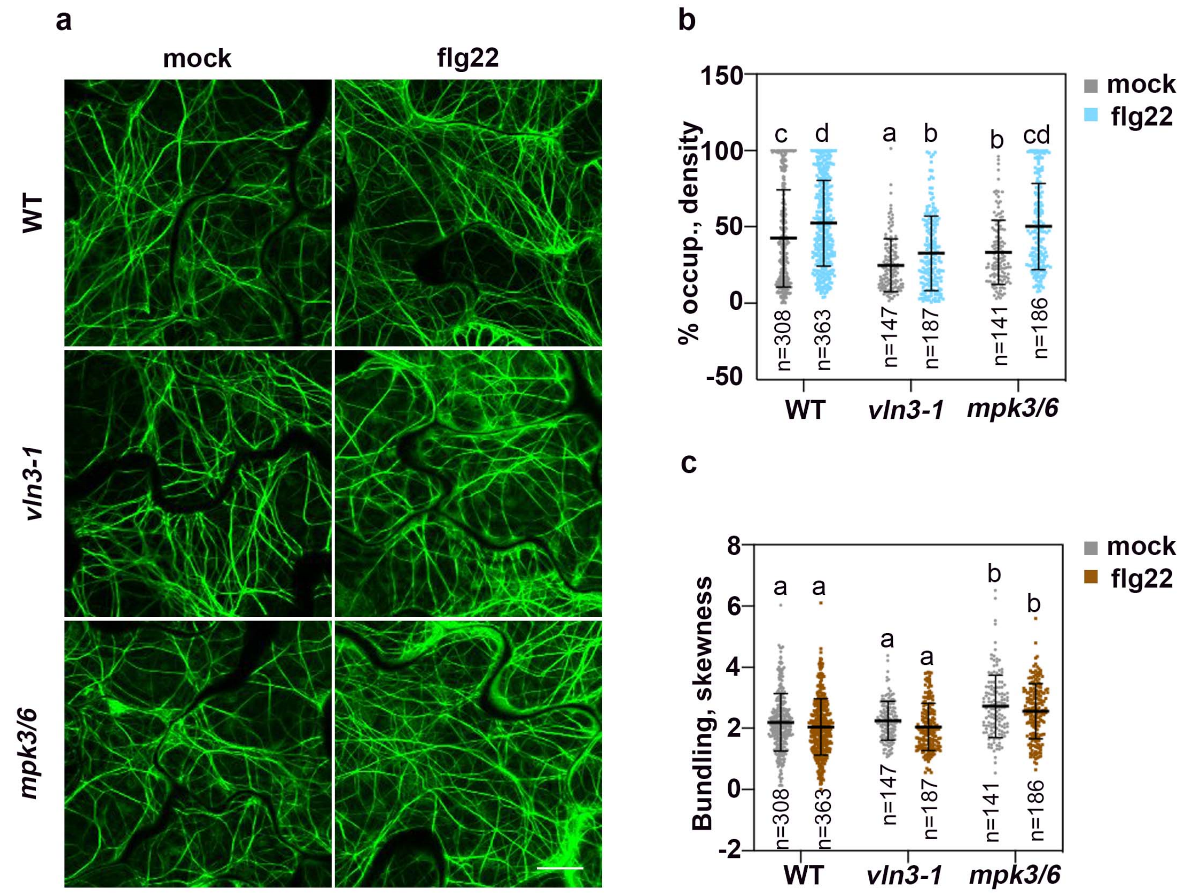


**Supplementary Figure 8. MPK3/MPK6 and VLN3 are not required for MAMP-triggered actin response in epidermal pavement cells.**

(a) Representative images of epidermal cells from WT and mutant rosette leaves treated with flg22. Scale bars = 10 µm. (b,c) Actin architecture was measured by density (b) and skewness (c) analyses on wild-type and mutant epidermal cells after treatment with flg22. Values given are means ± SD. The number of cells measured are indicated. Different letters indicate significant differences at P<0.05, as determined by two-way ANOVA with Tukey's multiple comparisons test. The exact p-values are provided in the Source Data file.
