# Supplementary material for: MPK3- and MPK6-mediated VLN3 phosphorylation regulates actin dynamics during stomatal immunity in Arabidopsis
Source: Nat Commun. 2021 Nov 9;12:6474. doi: 10.1038/s41467-021-26827-2 (PMC8578381; doi:10.1038/s41467-021-26827-2)
Supplement: Supplementary file 3 — Description of Additional Supplementary Files [file 41467_2021_26827_MOESM3_ESM.pdf]

Description of Additional Supplementary Files

File name: Supplementary Movie 1

Description: Time-lapse TIRF microscopy series of actin filaments in the presence of activated MPK3.

File name: Supplementary Movie 2

Description: Time-lapse TIRF microscopy series of actin filaments in the presence of VLN3.

File name: Supplementary Movie 3

Description: Time-lapse TIRF microscopy series of actin filaments in the presence of VLN3 phosphorylated by activated MPK3.

File name: Supplementary Movie 4

Description: Time-lapse TIRF microscopy series of actin filaments in the presence of VLN3S779A and activated MPK3.

File name: Supplementary Movie 5

Description: Time-lapse TIRF microscopy series of actin filaments in the presence of VLN3S779D.

File name: Supplementary Movie 6

Description: Time-lapse TIRF microscopy series of actin filaments in the presence of VLN3 and MKK5DD.

File name: Supplementary Movie 7

Description: Time-lapse images of actin filament dynamics in WT guard cells treated with mock.

File name: Supplementary Movie 8

Description: Time-lapse images of actin filament dynamics in WT guard cells treated with flg22.
