# Supplementary material for: MPK3- and MPK6-mediated VLN3 phosphorylation regulates actin dynamics during stomatal immunity in Arabidopsis
Source: Nat Commun. 2021 Nov 9;12:6474. doi: 10.1038/s41467-021-26827-2 (PMC8578381; doi:10.1038/s41467-021-26827-2)
Supplement: Supplementary file 12 — Reporting Summary [file 41467_2021_26827_MOESM12_ESM.pdf]

nature portfolio

Corresponding author(s): Jiejie Li

Last updated by author(s): Oct 16, 2021

Reporting Summary

Nature Portfolio wishes to improve the reproducibility of the work that we publish. This form provides structure for consistency and transparency in reporting. For further information on Nature Portfolio policies, see our Editorial Policies and the Editorial Policy Checklist.

Statistics

For all statistical analyses, confirm that the following items are present in the figure legend, table legend, main text, or Methods section.

n/a	Confirmed	
☐	☒	The exact sample size (*n*) for each experimental group/condition, given as a discrete number and unit of measurement
☐	☒	A statement on whether measurements were taken from distinct samples or whether the same sample was measured repeatedly
☐	☒	The statistical test(s) used AND whether they are one- or two-sided<br>*Only common tests should be described solely by name; describe more complex techniques in the Methods section.*
☒	☐	A description of all covariates tested
☒	☐	A description of any assumptions or corrections, such as tests of normality and adjustment for multiple comparisons
☐	☒	A full description of the statistical parameters including central tendency (e.g. means) or other basic estimates (e.g. regression coefficient) AND variation (e.g. standard deviation) or associated estimates of uncertainty (e.g. confidence intervals)
☐	☒	For null hypothesis testing, the test statistic (e.g. *F*, *t*, *r*) with confidence intervals, effect sizes, degrees of freedom and *P* value noted<br>*Give P values as exact values whenever suitable.*
☒	☐	For Bayesian analysis, information on the choice of priors and Markov chain Monte Carlo settings
☒	☐	For hierarchical and complex designs, identification of the appropriate level for tests and full reporting of outcomes
☒	☐	Estimates of effect sizes (e.g. Cohen's *d*, Pearson's *r*), indicating how they were calculated

Our web collection on statistics for biologists contains articles on many of the points above.

Software and code

Policy information about availability of computer code

Data collection	Microscopic images were collected using Carl Zeiss Zen software. The luminescence was recorded using a FlexStation3 (Molecular Devices).The phosphorylation signals were detected by a Typhoon 9410 phosphor imager. Peptide identification and phosphosites assignments were performed with the Proteome Discoverer software (version 1.4, Thermo Fisher).TIRF images were collected at 1- to 2-s intervals using ELYRA 7 (Carl Zeiss).
Data analysis	Images were analyzed using ImageJ/Fiji software (version 2.0.0-rc-68/1.52h). Data were analyzed using graphpad prism software.For correlation coefficient analyses, time-lapse VAEM series were cropped and analyzed using the built-in MATLAB function corr2 defined by Vidali et al. (Vidali, L. et al. Myosin XI is essential for tip growth in Physcomitrella patens. Plant Cell 22, 1868-1882 (2010)).

For manuscripts utilizing custom algorithms or software that are central to the research but not yet described in published literature, software must be made available to editors and reviewers. We strongly encourage code deposition in a community repository (e.g. GitHub). See the Nature Portfolio guidelines for submitting code & software for further information.

Data

Policy information about availability of data

All manuscripts must include a data availability statement. This statement should provide the following information, where applicable:

- Accession codes, unique identifiers, or web links for publicly available datasets
- A description of any restrictions on data availability
- For clinical datasets or third party data, please ensure that the statement adheres to our policy

The authors declare that all data supporting the findings of this study are included in the manuscript and its supplementary files are available from the corresponding author upon request. Source data are provided with this paper.

Field-specific reporting

Please select the one below that is the best fit for your research. If you are not sure, read the appropriate sections before making your selection.

☒ Life sciences ☐ Behavioural & social sciences ☐ Ecological, evolutionary & environmental sciences

For a reference copy of the document with all sections, see nature.com/documents/nr-reporting-summary-flat.pdf

Life sciences study design

All studies must disclose on these points even when the disclosure is negative.

Sample size	Sample size was determined for each experiment based on similar data reported in the scientific literature.
Data exclusions	No data were excluded from the analyses.
Replication	At least two or three independent biological repeats were done for all experiments.
Randomization	Data were randomly collected.
Blinding	All data collection and analysis were performed as double-blind experiments.

Reporting for specific materials, systems and methods

We require information from authors about some types of materials, experimental systems and methods used in many studies. Here, indicate whether each material, system or method listed is relevant to your study. If you are not sure if a list item applies to your research, read the appropriate section before selecting a response.

Materials & experimental systems

n/a	Involved in the study	
☐	☒	Antibodies
☒	☐	Eukaryotic cell lines
☒	☐	Palaeontology and archaeology
☒	☐	Animals and other organisms
☒	☐	Human research participants
☒	☐	Clinical data
☒	☐	Dual use research of concern

Methods

n/a	Involved in the study	
☒	☐	ChIP-seq
☒	☐	Flow cytometry
☒	☐	MRI-based neuroimaging

Antibodies

Antibodies used	Anti-FLAG (RM1002) (Beijing Ray Antibody Biotech), dilution factor: 1:2000<br>Anti-ACTIN (AC009) (ABclonal), dilution factor: 1:2000<br>Anti-GFP (RM1008) (Beijing Ray Antibody Biotech), dilution factor: 1:2000<br>Anti-MPK6 (A7104) (Sigma-Aldrich), dilution factor: 1:2000<br>Anti-p44/42 MAPK (4370) (Cell Signaling Technology), dilution factor: 1:2000<br>Anti-GST (RM1005) (Beijing Ray Antibody Biotech), dilution factor: 1:2000<br>Anti-HA (RM1004) (Beijing Ray Antibody Biotech), dilution factor: 1:2000<br>Anti-pS779 (Abmart), dilution factor: 1:1000
Validation	Anti-FLAG:http://www.rayantibody.com/index.php?c=product&a=type&tid=12<br>Anti-ACTIN:https://abclonal.com.cn/catalog/AC009<br>Anti-GFP:http://www.rayantibody.com/index.php?c=product&a=type&tid=12<br>Anti-MPK6:https://www.sigmaaldrich.com/catalog/product/roche/11814460001?lang=zh&region=CN<br>Anti-p44/42 MAPK:https://www.cellsignal.cn/products/primary-antibodies/phospho-p44-42-mapk-erk1-2-thr202-tyr204-d13-14-4e-xp-rabbit-mab/4370?site-search-type=Products&N=4294956287&Ntt=4370&fromPage=plp<br>Anti-GST:http://www.rayantibody.com/index.php?c=product&a=type&tid=12<br>Anti-HA:http://www.rayantibody.com/index.php?c=product&a=type&tid=12<br>Anti-pS779:http://www.ab-mart.com
